# A Multidisciplinary Approach in the Treatment of Tempromandibular Joint Pain Associated with Qat Chewing

**DOI:** 10.1155/2013/139419

**Published:** 2013-03-13

**Authors:** Mansoor Shariff, Mohammed M. Al-Moaleem, Nasser M. Al-Ahmari

**Affiliations:** ^1^Prosthodontic Department, College of Dentistry, King Khalid University, P.O. Box 3263, Abha 61471, Saudi Arabia; ^2^College of Dentistry, King Khalid University, P.O. Box 3263, Abha 61471, Saudi Arabia

## Abstract

Pain of the tempro-mandibular joint (TMJ) has a direct bearing to missing teeth and excessive physical activity. Consumption of qat requires chewing on the leaves to extract their juice for long hours. A 65-year-old male Yemeni patient, a Qat chewer, reported to the university dental hospital at King Khalid University complaining of pain in left temporomandibular joint with missing mandibular anterior teeth. A multidisciplinary approach for the overall treatment of the patient was decided. Initial treatment was the relief of patient's pain with the help of a night guard. This was followed by a fabrication of anterior FPD. The case was under maintenance and follow-up protocol for a period of 8 months with no complaint of pain discomfort.

## 1. Introduction

Qat-chewing habit in Yemen is widely spread and practiced by a majority of the populace [1]. Qat is the leaves of the shrub *Catha edulis* which are chewed like tobacco or used to make tea; it has the effect of a euphoric stimulant. Fresh qat leaves are usually chewed during social and cultural gatherings and held in the lower buccal pouch unilaterally in a bolus for long hours [2, 3]. Chewing Qat has been practiced for central stimulant effects; the pleasurable central stimulant properties of Qat are commonly believed to improve work capacity, during travelling, by students preparing for exams and counteract fatigue [4]. TMJ pain is the most common compliant seen among TMJ dysfunction patients especially Qat chewers. The pain is commonly originated from TMJ and masticatory muscle dysfunction. An occlusal appliance/a splint is a removable device, usually made of hard acrylic, that fits over the occlusal and incisal surfaces of teeth in one arch, creating precise occlusal contact with the teeth of the opposing arch [5]. It may be used for occlusal stabilization, for treatment of TMJ disorder, or to prevent wear of the dentition [6]. Qat was reported to cause dental attrition, staining of teeth, TMJ disorders (pain and clicking), cervical caries, and increased periodontal problems [4–7]. If signs and symptoms of occlusal abnormalities are present, therapy should be initiated prior to any permanent prosthetic treatment. Replacement of the missing teeth by FPD should be in harmony with the existing occlusal relationship [8]. This paper describes a sequence of treatment for a qat-chewing patient with pain in left side TMJ and missing lower anterior teeth.

## 2. Case Report

**Figure 1:**
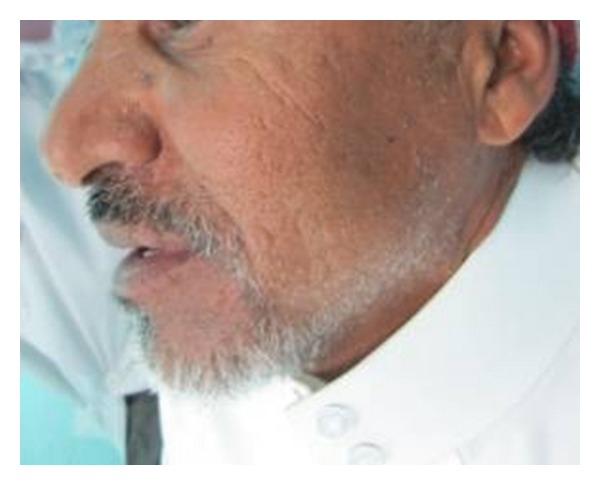
Left lateral view of the patient with hypertrophic masseter muscle.

**Figure 2:**
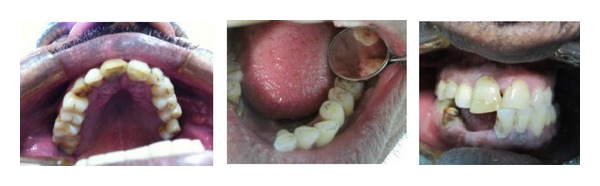
Intraoral view.

**Figure 3:**
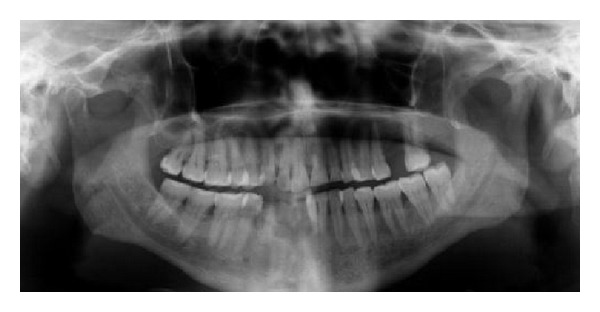
Preoperative OPG.

A 65-year-old male Yemeni patient presented to College of Dentistry, King Khalid University, dental clinics. The patient complained of chronic pain, dull in nature around his left ear and the left side of his face. The pain starts late in the night and early mornings. Reviewing his personal history, he habitually has been chewing Qat for over 30 years. The extraoral examination elicited pain in left masseter and TMJ. There was evidence of a hypertrophic left Masseter muscle (Figure 1). The intraoral examination showed server generalized attrition of all present teeth and a history of bruxism. Class I molar relationship and occlusal group function were observed. Teeth numbers 26, 41, 42, and 43 were missing (Figure 2). The radiographic interpretation revealed mild bone loss; flat anatomy of glenoid fossa and condyle on the left side was obvious. The position of the right condyle is slightly anteriorly bracing the articular eminence with normal anatomy of glenoid fossa (Figure 3).

**Figure 4:**
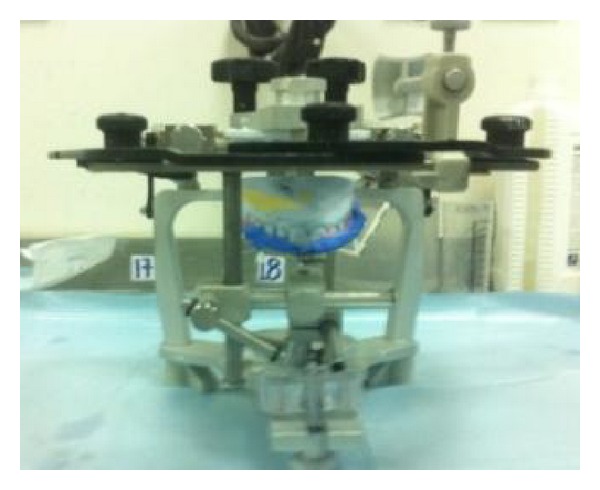
Face bow mounted on articulator.

**Figure 5:**
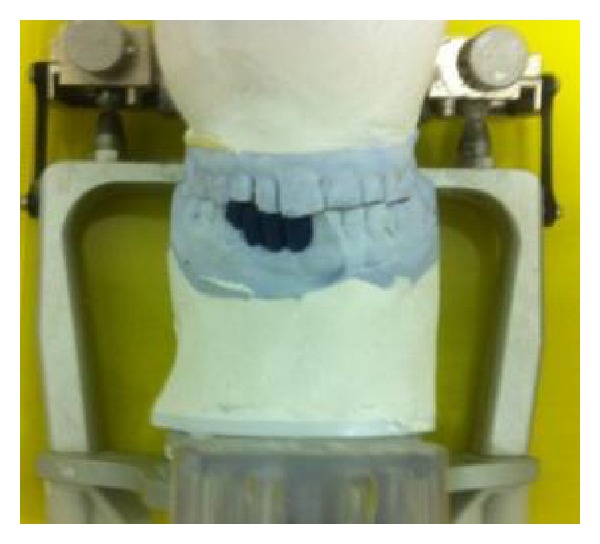
Diagnostic wax-up on mandibular arch.

**Figure 6:**
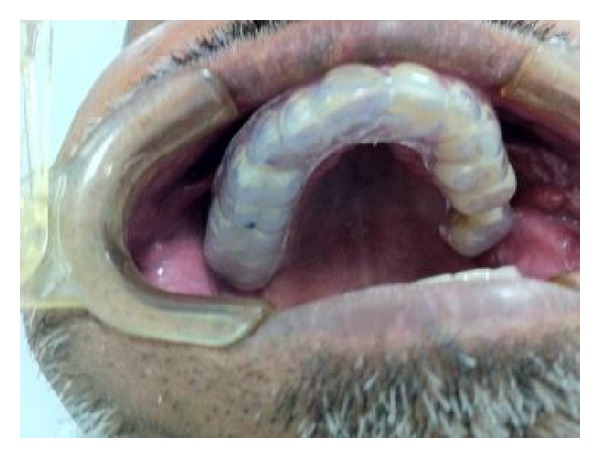
Soft splint on the maxillary teeth.

The treatment was initiated with periodontal therapy and oral hygiene instruction. Maxillary and mandibular impressions were made for diagnostic casts, which were mounted on semiadjustable Whip-Mix articulator (Waterpik Technologies, Fort Collins, Co, USA) after face bow transfer (Figure 4). The diagnostic wax-up was done in harmony with centric occlusion, protrusive and extrusive movements (Figure 5). Relaxation soft splint was constructed and given to the patient starting from 0.9 mm increasing to 2 mm (Figure 6). The premature contacts were identified and selective grinding was done intraorally with articulating paper 8 microns in thickness.

**Figure 7:**
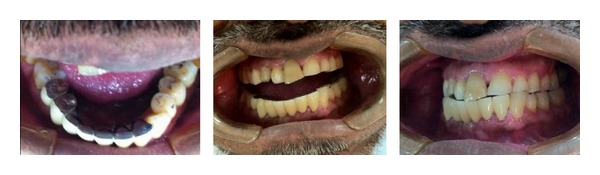
Definitive prosthesis after metal try-in and cemented FPD with metal occlusal surface.

After a one-month followup, there was considerable improvement in pain with only slight stiffness in the left TMJ in the early morning. After the second month, there was no pain and discomfort. Root canal treatment of tooth number 44 was done, followed by post and core buildup. A definitive fixed prosthesis was planned for replacing missing teeth number 43, 42, and 41. Provisional bridge was given to the patient in group function. After one month from the placement of the provisional bridge, definitive prosthesis was tried and cemented (Figure 7). The case was recalled after 3 and 6 months for maintenance phase. The patient was free of pain and discomfort.

## 3. Discussion

The harmony and sequence of the treatment are essential to be considered in this case. The preliminary assessment and relief of presenting symptoms, removal of etiological factors, RCT, prosthodontic treatment, and maintenance with follow-up program were needed for satisfactory outcome.

Before commencing any appliance therapy for a TMD, the clinician should be confident that the patient will benefit from the therapeutic approach. However, much controversy exists over the exact mechanism by which occlusal appliances reduce symptoms. Most conclusions are that they decrease muscle activity (particularly parafunctional activity) [5]. The prosthetic treatment of seriously damaged, endodontically treated teeth often requires an endodontic post as an additional retention element for core buildup prior to crown restoration [9]. Tooth number 44 was root-canal-treated; then fiber resin post was selected for reinforcing of the coronal as well as the radicular portion of the badly broken tooth. Mandibular canine lies outside the interabutment axis and forms the strongest point of force, so complex fixed partial denture with an additional abutment tooth in the arch was considered for replacement of the mandibular right anterior teeth. Tooth number 26 was extracted 13 years ago. Missing tooth should not be routinely replaced, especially in the presence of long-standing edentulous space with no drifting, elongation of adjacent or opposing teeth (stable occlusion). Porcelain abrades the opposing natural teeth because of their hardness; this causes a significant problem if the porcelain surface is roughened by occlusal adjustments [10]. Hence, metallic occlusal surface provides stable occlusal contacts without causing loss of the opposing tooth structure.

The clinical significance of this treatment is the relief of the pain by removing the cause with the replacement of missing teeth by a prosthesis in harmony with the existing occlusion.

## 4. Conclusion

A planned logical sequence was followed in the treatment of this case. Identifying the patient's chief complaint with symptomatic relief (occlusal therapy) followed by stabilization and correction of the occlusion with definitive fixed prosthesis and follow-up care was executed. The severe attrition and group function were considered during the construction and fabrication of fixed partial denture.
